# The Tendon Extracellular Matrix: From Hierarchical Architecture to Systemic Regulation and Therapeutic Translation

**DOI:** 10.3390/biom16101400

**Published:** 2026-09-25

**Authors:** Qianshuo Wang, Dingge Liu, Zhihua Zhang, Xin Zhang

**Affiliations:** Beijing Key Laboratory of Sports Injuries, Institute of Sports Medicine, Peking University Third Hospital, Beijing 100191, China; 2510301330@stu.pku.edu.cn (Q.W.); 2511110520@stu.pku.edu.cn (D.L.); 2311210463@stu.pku.edu.cn (Z.Z.)

**Keywords:** tendon extracellular matrix, collagen, non-collagenous protein, mechanobiology, regenerative medicine

## Abstract

The tendon extracellular matrix (ECM) is a complex and dynamic structure essential for transmitting force and ensuring musculoskeletal integrity. It is primarily composed of a hierarchy of collagen fibrils, whose assembly and stability are regulated by enzymatic and non-enzymatic cross-linking. Various non-collagenous proteins further modulate its organization and functional properties. The mechanical behavior of tendons is directly dictated by this specialized ECM, which in turn dynamically adapts to physiological loads. A continuous bidirectional dialogue exists between tenocytes and the surrounding matrix, crucial for tissue homeostasis. Furthermore, ECM remodeling is influenced by systemic and temporal factors, including circadian rhythms, hormones, aging, exercise, and genetics. Metabolic diseases, notably diabetes and hyperlipidemia disrupt ECM equilibrium by fueling chronic inflammation and pathological remodeling. Given its central role, the tendon ECM has emerged as a key target for innovative strategies in regenerative medicine, such as biologic therapies and the design of biomimetic scaffolds. This review synthesizes current knowledge on the composition, mechanobiology, regulation, and translational applications of the tendon ECM, providing an integrated perspective for advancing therapeutic approaches in tendinopathy and tendon repair.

## 1. Introduction

Tendons, the robust fibrous tissues connecting muscle to bone, are indispensable for transmitting force, facilitating movement, and ensuring skeletal stability [1]. Their biomechanical competence (exhibiting high tensile strength coupled with flexibility) is not an inherent property of the sparse resident tenocytes, but is primarily governed by the highly specialized extracellular matrix (ECM) that these cells produce and maintain [2]. The tendon ECM is predominantly composed of collagen. To date, seven fibril-forming collagen types have been identified (e.g., types I, II, III, V, XI, XXIV, and XXVII), with type I collagen constituting the majority of the tendon ECM. Other collagens, such as types II, VI and III, are present in smaller amounts but their roles are not negligible [3,4,5,6]. The importance of non-collagenous proteins within the tendon ECM is also increasingly recognized. Proteins such as elastin, the proteoglycans decorin, biglycan (BGN), lumican, fibromodulin, as well as lubricin and tenascin, along with their associated glycosaminoglycans (GAGs), are believed to contribute to the characteristic quasi-static and viscoelastic mechanical behavior of tendons and ligaments under tension, shear, and compression [7,8,9,10]. The collagen fibril is stabilized by intricate enzymatic and non-enzymatic cross-links, assembling into fibers, fascicles, and finally the whole tendon [11,12].

The core function of tendons is to efficiently transmit muscular force and store elastic energy, a capability fundamentally rooted in the highly specialized structure and dynamic properties of their ECM [13]. The ECM is not an inert structural scaffold but an actively responsive, homeostatic system for mechanosignal transduction and regulation [14]. Critically, the assembly and performance of the ECM are not pre-determined but are continuously shaped and optimized by physiological mechanical loading [2]. Furthermore, the ECM itself constitutes a functional stem cell niche. While cells remodel the ECM by sensing mechanical forces and chemical signals, the composition and physical properties of the ECM, in turn, instruct cell fate and function [15].

Tendinopathy is a prevalent musculoskeletal disorder characterized by progressive tendon structural degeneration and functional impairment, which can significantly compromise patients’ quality of life and athletic performance. While tendon pain is the most common clinical presentation, it is not universally present across all pathological states of tendinopathy. In fact, the emergence of pain is closely associated with the degree of neovascularization within the lesion site [1,16]. Tendinopathy demonstrates a high incidence within both the general population and specific occupational groups (e.g., athletes, manual laborers), establishing itself as a leading cause of chronic pain and functional disability [17,18]. The etiology and progression of tendinopathy constitute a complex, multifactorial process involving multiple interacting pathways. Risk factors are broadly categorized as extrinsic or intrinsic [1]. Extrinsic factors primarily encompass overuse (repetitive mechanical loading), improper training, and acute trauma. These impose mechanical stresses that surpass the tendon’s adaptive capacity, directly inducing collagen fiber micro-damage, matrix degradation, and inflammatory responses [19,20,21]. Intrinsic factors pertain to the individual’s physiological and pathological state, including aging (associated with altered collagen cross-linking and diminished cellular activity), genetic predisposition, sex-related differences (e.g., influences of estrogen levels), and increasingly recognized metabolic diseases such as type 2 diabetes and hyperlipidemia. These metabolic disorders not only independently elevate tendinopathy risk but also profoundly disrupt tendon ECM homeostasis, mechanical properties, and repair capacity. The process takes effect through molecular mechanisms that promote advanced glycation end-product (AGE) accumulation, oxidative stress, and chronic low-grade inflammation [22,23,24,25,26,27,28]. Therefore, a comprehensive understanding of the epidemiology of tendinopathy and its multi-layered determinants, particularly the central roles of metabolic dysregulation and inflammatory signaling, is essential for developing targeted preventive and therapeutic strategies.

The repair of tendon injuries and degenerative pathologies remains a significant clinical challenge, largely due to the impaired ECM’s limited capacity for spontaneous restoration of its highly ordered architecture and superior mechanical properties [29]. Advances in understanding the composition, function, and remodeling dynamics of the tendon ECM in disease states are driving a fundamental shift in regenerative medicine strategies—from traditional passive support (e.g., suturing, immobilization) towards actively targeting and modulating the ECM [30,31]. This interdisciplinary frontier, integrating biomaterials science, molecular biology, and tissue engineering, aims to reactivate or potentiate the tendon’s endogenous repair mechanisms through multimodal interventions [32,33].

In this narrative review, we will explore the intricate composition and hierarchical architecture of the tendon ECM, with a focus on its collagenous network, regulatory non-collagenous proteins, and critical cross-linking mechanisms. We will elucidate the dynamic, bidirectional interactions between the ECM and resident tendon cells that underpin tissue homeostasis and mechano-adaptation. From a translational perspective, we will analyze the disruptive impact of systemic factors (such as metabolic disease, aging, exercise, circadian rhythm and genetic predisposition) on ECM integrity, and evaluate emerging therapeutic strategies. This includes the potential of ECM-derived biologics and the rationale for designing bioinspired, functional composite scaffolds for regeneration. Finally, we will discuss the implications of ECM-centric approaches for the clinical management of tendinopathies and tendon injuries.

## 2. Composition of the Tendon ECM

The following description of tendon ECM composition and hierarchical structure refers primarily to the midportion of tendons.

### 2.1. Structural Organization of Tendon Across Scales

Tendons are highly ordered, multiscale connective tissues. Their mechanical competence derives as much from their organizational architecture as from the molecular composition of their extracellular matrix. At the organ level, a tendon is enveloped by a paratenon or, in certain anatomical locations, a synovial sheath. The outermost connective tissue layer, the epitenon, is continuous with the endotenon. the endotenon is a loose areolar connective tissue that invests individual fascicles and houses the tendon′s blood vessels, lymphatics, and nerves [34]. The tendon proper is subdivided into fascicles (secondary bundles, approximately 100–500 µm in diameter in human tendon), which are in turn composed of primary fascicles and collagen fibers. At the gross level these have been described as primary, secondary, and tertiary bundles aligned nearly parallel to the tendon long axis [34,35].

Crucially, fascicles are not bound by a rigid matrix but are separated by the interfascicular matrix (IFM). IFM is a compliant, proteoglycan rich compartment that is selectively enriched in elastin and lubricin (PRG4) and that permits sliding and elastic recoil between adjacent fascicles [36,37]. Within each fascicle, collagen fibrils (roughly 50–500 nm in diameter and frequently showing a bimodal size distribution) are assembled from microfibrils and tropocollagen molecules and display the characteristic approximately 67 nm D-period banding [35,38].

Tenocytes, the principal resident cells, are sparse and arranged in parallel rows between the collagen fibers, a cellular organization dictated by the surrounding matrix architecture [34]. The tendon's structure is also regionally heterogeneous: whereas the mid-substance is dominated by parallel type I collagen, the myotendinous junction exhibits interdigitating muscle–tendon interfaces that amplify contact area, and the osteotendinous enthesis transitions through a graded fibrocartilaginous zone that dissipates stress across the mechanically dissimilar muscle–tendon–bone continuum [39].

### 2.2. Collagen

#### 2.2.1. Collagen Types

Healthy tendons primarily function to transmit muscle-generated force to bones while imparting viscoelasticity to tissues. Their ECM is a sophisticated structure (Figure 1) [2]. Collagen is a vital component of the tendon ECM and is the key source of tendon mechanical strength. It is crucial for the mechanisms of force transmission, strength, and deformation within tendons [40]. As the core component of tendons, collagen accounts for 65–80% of tendon dry weight [2,34]. Type I collagen predominates, constituting approximately 95% of total tendon collagen. It comprises three α chains (two α-1 chains and one α-2 chain) arranged in a triple helix structure, establishing the foundation for tendon mechanical properties [21,34,41]. Additionally, type III collagen constitutes about 5%, typically forming finer, more irregularly arranged fibers primarily distributed in the tendon endomysium and epimysium. Type V collagen is embedded within the core of type I collagen fibers, regulating fiber diameter. Other collagen types (e.g., II, VI, IX, X, XI) are present in minimal quantities, predominantly concentrated in the fibrocartilaginous regions at bone attachment sites, where they help mitigate stress concentration at hard tissue interfaces [2,6,42]. Although present in lower concentrations, other types of collagen also play a role in regulating the homeostasis of the tendon ECM. Deficiency of collagen VI may impair the mechanical response of tendon fibroblasts in Ulrich congenital muscular dystrophy [6]. Ablation of collagen XII disrupts the articular ECM, leading to patellar subluxation [43]. Functioning as a key integrator, collagen XII regulates matrix assembly not only by binding to collagen fibers in the extracellular space but also by constructing pericellular matrix bridges, thereby coordinating tendon cell morphology, alignment, and communication networks [44]. During postnatal development, collagen XI (encoded by the Col11a1gene) is essential for determining normal tendon structure and mechanical properties through its precise regulation of collagen fibril nucleation and assembly [45,46]. The role of collagen V in tendon healing exhibits a dose-dependent “Goldilocks effect.” Importantly, the impaired healing observed in conventional collagen V knockout models appears to originate primarily from pre-existing developmental matrix defects rather than from the repair process itself [47].

#### 2.2.2. The Hierarchical Structure of Collagen

The mechanical function of tendons relies on the precise hierarchical structure of collagen from the molecular to the tissue level [48]. Collagen is hierarchically organized in the form of protofibrils, fibrils, and fibrillar bundles [40,49]. Tendon cells synthesize the procollagen precursor, which is cleaved to form tropocollagen molecules. These tropocollagen molecules first assemble into a stable triple-helix structure—one of the strongest protein structures in the body. Subsequently, they assemble into collagen fibrils through non-covalent interactions, which are further stabilized by covalent cross-linking. Fibrils further aggregate into fibers, fibers into fascicles, ultimately constituting the entire tendon [50,51,52].

#### 2.2.3. Enzymatic Cross-Linking of Collagen

The network structure of collagen fibers within their ECM plays a significant role in the mechanical function of tendons, and collagen cross-linking represents a key biochemical modification determining the stability and mechanical properties of this network. Cross-linking mechanisms are primarily categorized into enzymatic and non-enzymatic types [2,40,53]. The formation of enzymatic cross-links is a sequential two-compartment process initiated by intracellular post-translational modification. Prior to procollagen secretion, lysyl hydroxylases (encoded by PLOD genes) catalyze the hydroxylation of specific lysine residues within procollagen chains in the rough endoplasmic reticulum. The resulting hydroxylysine residues serve as essential substrates for subsequent extracellular cross-link formation, and their modification level directly shapes the chemical type and stability of final cross-links [54]. Enzymatic cross-linking is predominantly catalyzed by lysyl oxidase (LOX) and its analogues, where LOX oxidizes specific lysine and hydroxylysine residues within the telopeptide domains of collagen molecules into reactive aldehydes. These aldehydes react with amino acids in the triple-helix regions of adjacent molecules, initially forming immature bivalent cross-links that subsequently spontaneously polymerize into mature trivalent cross-links [53,55,56]. This progression shifts the cross-link network from a reversibly dynamic state that supports stress relaxation to an irreversibly stable state that confers long-term tissue strength and rigidity. Enzymatic cross-linking occurs within collagen fibrils, linking adjacent collagen molecules, primarily through connections between telopeptidyl and triple-helical domains. This inhibits molecular sliding, increases fibril stiffness, and consequently enhances the strength and rigidity of tendon tissue [57,58]. Beyond mechanical properties, the density and maturity of enzymatic cross-links are a critical determinant of collagen susceptibility to proteolytic degradation by matrix metalloproteinases (MMPs). Mature trivalent cross-links introduce steric hindrance that blocks MMP access to cleavage sites within the collagen triple helix, thereby modulating ECM turnover kinetics and maintaining long-term tissue homeostasis [59]. The successful implementation of enzymatic cross-linking is highly dependent on the structural context of the collagen matrix. The “timely” maturation of hierarchical architecture creates the necessary conditions for the formation of functional cross-links. Studies indicate that the addition of exogenous LOXL2 is most effective during a specific window: treatment at the stage when collagen has assembled into larger, more ordered fibrillar structures (week 4) significantly enhances the accumulation of mature pyridinoline cross-links and substantially increases the tissue tensile modulus to levels comparable to native immature tissue. In contrast, intervention applied too early (week 0, unorganized collagen) or during initial fibril formation (week 2) yields limited benefit [60].

#### 2.2.4. Non-Enzymatic Cross-Linking of Collagen

Another significant cross-linking mechanism is non-enzymatic cross-linking. Non-enzymatic reactions between proteins or lipids and sugars, such as glucose and its derivatives, generate advanced glycation end products (AGEs). AGEs form irreversible covalent cross-links with adjacent proteins (collagen fibers), leading to their long-term accumulation in tissues with slow metabolism, such as tendons [61]. AGEs closely associated with tendon cross-linking include pentosidine (a pentose-derived AGE), methylglyoxal-lysine dimers, malondialdehyde-lysine adducts, carboxymethyl-lysine, and carboxyethyl-lysine [62,63,64]. The accumulation of AGEs in the tendon ECM (primarily composed of type I collagen) directly alters tendon physical and mechanical properties through non-enzymatic cross-linking formation [65]. Cross-links formed by ribose-derived AGEs (Ribo-AGEs) and methylglyoxal-derived AGEs (MGO-AGEs) on collagen reduce inter-fibrous sliding capacity and decrease tendon viscoelasticity. This directly leads to diminished tendon toughness and increased brittleness [61,66,67]. Cross-linking simultaneously increases the failure stress of tendon but reduces its toughness, making it more susceptible to injury under load [61].

### 2.3. Non-Collagenous Components

In the ECM of tendons, non-collagenous components play a significant role [7]. Elastin resists deformation along the fiber orientation, regulates shear and transverse mechanical properties, and regulates energy storage and dissipation [68,69,70]. Although relatively low in abundance compared with collagen, elastic fibers are critical for tendon connective tissue homeostasis. They act synergistically with the dominant collagen fiber system. Collagen provides high tensile stiffness for force transmission, while elastic fibers confer reversible extensibility, enable rapid elastic recoil following tensile deformation, and dissipate mechanical energy during cyclic loading to protect tendon tissue from fatigue injury. In addition, the elastic fiber network provides structural guidance for tenocyte alignment and participates in the regulation of perifascicular matrix remodeling [2]. Proteoglycans and glycosaminoglycans regulate collagen fiber formation, significantly influencing the quasi-static mechanical properties of tendons [71,72,73]. Among proteoglycans, small leucine-rich proteoglycans (SLRPs) serve as crucial components of the ECM. They are not only structural elements of tendons but also key regulators in tendon development, homeostasis, and post-injury repair. SLRPs include core proteoglycans such as decorin (DCN), BGN, fibromodulin (FMOD), and lumican (LUM) [9]. DCN is the most abundant SLRP in tendons, promoting tendon cell proliferation while limiting lateral fusion of fibers [74,75,76]. BGN exhibits peak expression in embryonic tendons and drives the differentiation of tendon stem cells (TSPCs) into tendon cells. Contrary to DCN, BGN inhibits tendon cell proliferation [77]. Research indicates that BGN plays a more critical and active role than DCN in maintaining tendon ECM homeostasis, rather than merely exerting a secondary regulatory function [78]. Furthermore, a clear synergistic and hierarchical relationship exists between BGN and Collagen VI. While BGN assists in stabilizing collagen fiber architecture and contributes to maintaining tendon structural-mechanical properties, its dominant effects on cell morphology, fiber assembly, and viscoelasticity are weaker than those of Collagen VI [79]. FMOD is an indispensable component of the TSPC niche. It binds to collagen and enzymes to form a LOX-FMOD-collagen ternary complex. The complex guides collagen molecules toward ordered cross-linking and assembly, forming regular fiber structures [80]. LUM restricts lateral growth of collagen fibers. In the absence of FMOD, LUM expression is upregulated to compensate, but its role in regulating tendon mechanical strength is less pronounced than that of FMOD [81]. Fibulin-4 may functionally interact with SLRP, primarily conferring mechanical strength to tendons by regulating collagen fibril assembly and size distribution [82]. Normal Cartilage Oligomeric Matrix Protein (COMP) forms pentamers and interacts with other matrix proteins (such as type I collagen) to jointly maintain the normal structure and homeostasis of the ECM. However, pathogenic mutations (such as p.V66E) disrupt COMP oligomerization, induce endoplasmic reticulum stress, trigger inflammation and fibrosis, and alter the composition and structure of the ECM (e.g., by forming ectopic microfibrils) [83].

## 3. Mechanical Forces and Tendon ECM

### 3.1. Mechanical Properties of the Tendon ECM

In the ECM of tendons, fibers are arranged in parallel to withstand unidirectional, high-rate, high-load mechanical stress, thereby achieving extremely high tensile strength [84,85]. Highly oriented type I collagen fibers are pivotal to the’ exceptional mechanical properties of tendon. Research indicates that the tensile mechanics of tendon ECM fundamentally depend on the mechanical continuity of its collagen fibers [86]. Collagen fibers are not primarily designed to resist compressive loads. Cross-links between collagen fibers mediate shear deformation, enabling fibers to resist compression collectively [87]. Furthermore, collagen and elastic fibers play distinct yet complementary roles in tendon elastic behavior. Collagen primarily provides stiffness and elastic recovery, while elastin confers viscoelasticity and safe extensibility under large deformations. Their synergistic action ensures tendons efficiently transmit forces during daily activities while preventing structural damage [88,89]. In mature tendon homeostasis, biglycan serves as a central regulator of ECM mechanical properties. Its absence leads to comprehensive declines in tendon stiffness, strength, dynamic load capacity, and viscoelastic behavior, accompanied by unstable rearrangement of the collagen fiber network under stress. In contrast, the more abundant decorin plays a limited role in the mechanical homeostasis of mature tendons. The surrounding/pericellular matrix may maintain the niche for tendon stem cells and regulate growth-active factors [78]. In the tensile mechanical behavior of the Achilles tendon, the degree of collagen fiber disorganization shows moderate to strong correlation, while the content of AGEs shows no statistically significant association [90].

### 3.2. Effects of Mechanical Forces on the Tendon ECM

Moreover, mechanical loading not only influences the strength of the ECM but also actively guides its assembly, remodeling, and determines its long-term stability, playing a role in tendon healing [91]. The tendon ECM is not a passive load-bearing structure but a dynamic, mechanochemically responsive system [3]. Through these extracellular mechanisms, mechanical forces direct the precise assembly, alignment, and orientation of collagen fibers, thereby conferring anisotropic mechanical properties to tendons [92]. The cross-linking and glycosylation levels within the tendon collagen network are not static but undergo dynamic, reversible chemical changes in response to mechanical stretching. The reversibility of immature bivalent imine cross-links constitutes a key chemical mechanism enabling tendon stress relaxation, elastic recovery, and avoidance of plastic deformation [93]. Physiological mechanical loading in vivo actively optimizes tendon ECM composition and structure by upregulating type I/III collagen and elastin synthesis, and tightly regulates matrix metalloproteinase-mediated ECM remodeling. [94]. In this process, MMP-1functions as the primary protease that initiates degradation of native fibrillar type I collagen, whereas MMP-2 primarily targets denatured collagen and basement membrane components to facilitate subsequent remodeling steps. Notably, MMP-1 expression is inversely regulated by mechanical tensile loading. Static tensile stress inhibits MMP-1 expression in tendon cells in a dose-dependent manner, while load deprivation significantly upregulates MMP-1 transcription and protein production via a cytoskeletally based mechanotransduction pathway [95]. Furthermore, mechanical loading activates tendon cells via the β1 integrin-integrin-linked kinase (ILK)-AKT-mTOR signaling axis, thereby upregulating type I and III collagen synthesis [96]. However, studies also indicate that the loading effect exhibits significant temporal dependence. While mechanical loading markedly enhances the mechanical properties and tissue maturation of the early-stage (1–2 weeks) ECM, this effect diminishes over time and becomes insignificant by week 4 [97].

### 3.3. Effects of Mechanically Mediated Molecular Pathways on the Tendon ECM 

Additionally, the mechanical properties of the tendon ECM are closely linked to several molecular pathways. The mechanosensitive ion channel Piezo1 serves as a central regulator of tendon mechanosensing and ECM mechanical properties, converting mechanical forces into biological responses and directly modulating the composition and mechanical characteristics of the tendon ECM [98]. Specifically, Piezo1 senses shear stress generated by collagen fiber sliding, activates calcium influx, and subsequently upregulates tendon-specific transcription factors Mohawk (Mkx) and Scleraxis (Scx). This activation triggers the downstream calcineurin-NFAT (nuclear factor of activated T-cells) pathway to promote collagen synthesis and ECM assembly [99,100]. Furthermore, increased Ca^2+^ signaling via the voltage-gated calcium channel Cav1.2 accelerates collagen fiber assembly and upregulates the expression of myostatin, matrix metalloproteinases (MMPs), and ECM glycoproteins, thereby enhancing tendon structural and mechanical properties [101]. Cellular mechanical tension also regulates tendon ECM homeostasis through an epigenetic mechanism mediated by the Yap/Taz axis. Under normal tension, Yap/Taz actively suppresses the expression of catabolic genes such as MMPs by maintaining the open state of specific chromatin regions. When tension is lost, the attenuation of Yap/Taz signaling leads to the closure of these regions, releasing the inhibition on the catabolic program and initiating ECM degradation [102]. Furthermore, the tendon ECM plays a key role in the pathological process of heterotopic ossification following injury. Polycystin-1 (PC1) acts as a mechanosensor that activates the Taz signaling axis, driving stem cells toward abnormal differentiation and orchestrating the ordered assembly of the ECM, thereby collectively promoting heterotopic bone formation. Targeting the “mechanical force-PC1-Taz-ECM” axis represents a potential new strategy for preventing post-traumatic heterotopic ossification (Figure 2) [103].

Mechanotransduction in tendon can be understood as a sequential cascade. Mechanical cues are sensed at the cell membrane, propagated through the cytoskeleton, and converted into transcriptional programmes that govern ECM synthesis and remodelling. In addition to Piezo1, which converts shear and tensile stress into Ca^2+^ influx and tunes tendon stiffness, mechanical loading is detected by integrin-based adhesions [99]. These adhesions cluster upon force application and recruit focal adhesion kinase (FAK) and Src, initiating downstream signalling [41,104]. The primary cilium–polycystin complex and additional stretch-activated channels have also been implicated. However, their contribution in tendon remains less firmly established and warrants cautious interpretation [105]. Downstream of these sensors, force is propagated along the actin cytoskeleton and transmitted by molecular clutch proteins together with RhoA/ROCK-mediated contractility, while Ca^2+^ influx activates calcineurin–NFAT signalling and integrin-linked kinase (ILK) engages the Akt–mTOR axis [104,105].

Several signalling pathways convert these proximal events into changes in ECM gene expression. Tension-dependent maintenance of tendon identity requires TGF-β/Smad2/3 signalling. Smad3 physically interacts with the tendon transcription factors Scx and Mkx, and loss of Smad3 reduces tendon tensile strength and disrupts collagen organization [106,107]. By contrast, Wnt/β-catenin signalling acts as a brake on tenogenic gene expression. It suppresses Scx, Mkx and tenomodulin and antagonises TGF-β-driven tenogenic induction [108]. The Hippo pathway effectors YAP and TAZ function as epigenomic guardians of matrix homeostasis. Loss of cytoskeletal tension reduces chromatin accessibility at YAP/TAZ target loci and derepresses matrix-degrading enzymes, whereas constitutively active YAP preserves the matrix programme [102]. In parallel, Ca^2+^ entry through L-type channels such as CaV1.2 promotes tendon hypertrophy and fibrillogenesis [101]. These cascades converge on the tendon transcription factors Scx, Mkx and Egr1, which drive expression of type I collagen and other matrix constituents [104].

The net output depends on the nature of the load. Physiological tensile loading is predominantly anabolic, upregulating collagen synthesis and stiffening matrix components. Whereas excessive or unaccustomed strain, or a shift from tensile to compressive loading, redirects tenocytes towards catabolic and non-tenogenic programmes, which can activate inflammatory NF-κB/IKKβ signalling, an established driver of degenerative tendinopathy [104,109]. Thus, the same core mechanotransduction machinery can either preserve or degrade the tendon ECM depending on loading context. This helps explain why both underload and overload predispose to tendon pathology.

## 4. The Microenvironment of Tendon ECM and Its Interaction with Cells

The relationship between tendon cells and collagen fibers is both structurally and biochemically fundamental. Tendons are hypocellular tissues. Their resident cells, immature tenoblasts and mature tenocytes, are elongated, spindle-shaped cells whose long axes lie parallel to the collagen fibers [110]. Tenocytes are not randomly dispersed. They are arranged in rows between collagen fiber bundles, extending broad, flat lateral cytoplasmic processes that wrap around adjacent collagen bundles, as well as long, thin longitudinal processes that reach toward more distant cells [111]. Through these processes, neighbouring tenocytes are coupled by gap junctions composed principally of connexin 43 (and connexin 32 between cell bodies). This forms a three-dimensional cellular network that permits intercellular passage of ions and small signalling molecules below approximately 1 kDa, and thereby distributes mechanical information throughout the tissue [112]. At the cell surface, a thin pericellular matrix enriched in type VI collagen, decorin and fibronectin anchors the tenocyte membrane to the surrounding fibrillar network. Integrin-based adhesions (including α1β1, α2β1, α5β1 and αvβ3) link to the actin cytoskeleton through talin, vinculin and paxillin, transducing force across the cell–matrix interface [96,111].

This relationship is bidirectional. Tenocytes not only sense the collagen network but actively construct and maintain it. Collagen fibrillogenesis is a cell-directed process in which procollagen is secreted through specialised plasma membrane protrusions termed fibripositors. These structures are coaligned with the tendon axis and thereby specify the characteristic parallel organisation of tendon collagen [84]. Subsequent fibril alignment and transport depend on actin filaments and non-muscle myosin II, directly coupling cytoskeletal dynamics to matrix architecture [113,114]. Conversely, because tenocytes are mechanically tethered to the fibrils they surround, applied loads are transmitted to the cells through these adhesions and the pericellular matrix rather than uniformly. The local strain experienced by a tenocyte is substantially lower than the strain applied to the whole fascicle, reflecting the crimped and markedly non-homogeneous strain field within the fascicle [115]. This cell–matrix mechanical coupling establishes a tensional set point that determines whether tenocytes mount an anabolic or catabolic response, providing the structural basis for the mechanotransduction cascades.

As a specialized tenogenic stem cell niche, the ECM regulates TSPCs and mature tenocytes via both structural components and physical mechanical cues. BGN and FMOD function as niche organizers that fine-tune bone morphogenetic protein (BMP) signaling intensity, thereby precisely maintaining TSPC self-renewal and guiding tenogenic lineage commitment [116,117]. The highly ordered fibrillar architecture of native tendon ECM further provides topographical cues that direct TSPC morphological alignment and tenogenic differentiation. Aligned matrix nanotopography induces spindle-shaped cell morphology and upregulates tendon-specific gene expression, whereas disorganized topography promotes aberrant osteogenic differentiation [118].

In parallel, the ECM serves as the primary transducer of mechanical loading to tendon cells, with the actin cytoskeleton functioning as the central mechanosensory hub. Physiological tensile forces are transmitted intracellularly via integrin-mediated cell–matrix adhesions, and cytoskeletal tension dynamics activate downstream anabolic cascades including the TGF-β-SMAD3 pathway, which upregulates synthesis and ordered assembly of key ECM components such as thrombospondin-4b (TSP4B) [119]. This actin cytoskeleton-dependent mechanotransduction axis also represents a key therapeutic target. Studies demonstrate that cytoskeleton-nuclear envelope mechanical coupling regulates downstream transcriptional effectors including YAP/TAZ, which underlies the therapeutic effects of physical interventions such as extracorporeal shock waves and collagen-based medical devices [120].

Soluble signaling molecules within the ECM microenvironment provide another critical layer of homeostatic regulation, acting in concert with structural and mechanical cues to balance ECM synthesis and degradation. The secreted soluble glycoprotein CTHRC1 acts as a soluble ligand that binds EGFR and activates the downstream MAPK pathway, enhancing TSPC anabolic activity and promoting high-quality ECM synthesis and ordered assembly [121]. TSPC-derived exosomes also serve as paracrine soluble mediators that balance ECM turnover by downregulating MMP-3 and upregulating its inhibitor TIMP-3, alongside increasing the expression of tendon-specific genes such as type I collagen and tenomodulin [122]. Under physiological tensile stress, this soluble regulatory network cooperates with mechanical signaling to suppress catabolic and pro-inflammatory pathways to further stabilize ECM homeostasis [123].

## 5. Factors Influencing Tendon ECM

### 5.1. Circadian Rhythm

ECM microenvironment is dynamic and undergoes changes across multiple timescales [124]. In tendons, ECM homeostasis is regulated by the circadian clock, which orchestrates the rhythmic synthesis and degradation of collagen through the temporal control of secretory pathways [125]. While the transcription of type I collagen genes (Col1a1, Col1a2) itself is not rhythmic, the protein levels of collagen I α1/α2 chains and their C-terminal propeptides exhibit pronounced circadian oscillations, with synthesis peaking at night. This post-transcriptional regulation is achieved through the rhythmic expression of key secretory pathway components, including the endoplasmic reticulum translocon SEC61, the endoplasmic reticulum export factor TANGO1, the Golgi-associated protein PDE4D, the post-Golgi trafficking component VPS33B, and the collagenase cathepsin K (CTSK) [126]. Furthermore, the circadian clock pre-adapts to nightly collagen synthesis peaks by rhythmically upregulating molecular chaperones such as BiP, thereby preventing endoplasmic reticulum stress and ensuring efficient collagen processing and secretion [127].

Within tendons, the endogenous clock drives the rhythmic expression of Mmp14, which encodes membrane type I-matrix metalloproteinase (MT1-MMP). MT1-MMP cleaves ECM components (fibronectin), and through mechanisms potentially independent of its direct collagenolytic activity, prevents excessive collagen cross-linking. This activity maintains a dynamic ECM microenvironment, which in turn provides physical and biochemical signals necessary to sustain robust cellular circadian rhythms, forming a critical feedback loop [128]. The circadian clock also influences tendon ECM composition by regulating the Grem2/BMP signaling pathway. The clock-controlled expression of the BMP antagonist Gremlin-2 (Grem2) creates a temporal gate that periodically inhibits BMP-Smad1/5 signaling, thereby protecting tendons from pathological osteogenic differentiation and ectopic calcification. Disruption of this circadian mechanism is a key contributor to the pathogenesis of calcific tendinopathy [129].

Based on the discovery that tendon ECM synthesis exhibits circadian rhythms, the concept of “chronotherapy” has been proposed for clinical application. For instance, scheduling exercise rehabilitation or therapeutic interventions during the daily peak of collagen synthesis may yield superior outcomes [125]. Furthermore, core circadian pathways represent promising therapeutic targets. The cryptochrome circadian regulator (CRY) agonist KL001 stabilizes CRY proteins and abolishes circadian rhythms in wild-type cells, while the REV-ERB agonist SR9009 attenuates cellular rhythms and reduces collagen fiber deposition. These findings indicate that pharmacologically targeting the circadian machinery can bidirectionally regulate collagen homeostasis [126,130]. This approach offers a potential strategy for treating ECM disorders such as tendon fibrosis and ectopic calcification resulting from circadian disruption.

### 5.2. Hormones

The effects of estrogen on tendon biology are complex and highly dependent on its concentration, physiological state, and tissue loading conditions [131]. It may promote collagen synthesis and increase collagen content in the tendon ECM, potentially through indirect regulation of the insulin-like growth factor-1 (IGF-1) signaling pathway [132]. Furthermore, estrogen appears crucial for reconstructing a normal, gradient-structured ECM with biomechanical cushioning properties at the healing site, particularly within the fibrocartilaginous zone. Estrogen deficiency could lead to misdirected ECM maturation, thereby preventing the formation of an optimal functional enthesis. It may also indirectly disrupt normal ECM synthesis and assembly at the tendon-bone interface by altering the local microenvironment [133]. Concurrently, estrogen is implicated in regulating collagen degradation and remodeling by modulating the expression of MMPs [134]. Supporting this, in vitro experiments using rat Achilles tendon cells have shown that estrogen deficiency reduces type I collagen production while increasing the expression of type III collagen and MMP-13, suggesting an impaired intrinsic healing capacity [135].

The biological effects of estrogen are concentration-dependent. Physiologically high concentrations can potently inhibit LOX activity, likely through post-translational regulation. This inhibition reduces collagen fiber cross-linking, leading to decreased tendon stiffness and increased joint laxity. This mechanism explains the heightened risk of ligamentous injuries observed in women during the ovulatory phase when estrogen levels peak [136]. Consistent with this, in young women, endogenous estrogen may suppress overall collagen synthesis and reduce tissue stiffness, contributing to increased injury risk during specific menstrual cycle phases, such as the late follicular phase [137].

Interestingly, despite a potentially higher risk for certain anterior cruciate ligament injuries, females may exhibit a lower risk of Achilles tendon rupture compared to males. Proteomic analysis via mass spectrometry revealed no significant sex-based differences in the abundance of major tendon structural proteins. However, three ECM proteins implicated in tissue repair, inflammation, and fibrosis were markedly elevated in females: fibronectin (~140% increase), periostin (~182% increase), and tenascin C (~89% increase) [138]. The estrogen-mediated reduction in tendon stiffness may enhance tissue elasticity, allowing for greater strain absorption during muscle contraction. This adaptive mechanism could serve as a protective factor, potentially explaining why female athletes demonstrate lower rates of certain muscle strain injuries compared to males [139].

Beyond estrogen, signaling through the growth hormone/insulin-like growth factor-I (GH/IGF-I) axis critically influences ECM. In mouse Achilles tendons, insufficient GH/IGF-I signaling severely disrupts collagen fibril assembly and diameter distribution, leading to an aberrant ECM ultrastructure. Conversely, while excessive activation of this axis strongly upregulates the expression of collagen and related genes, it has a comparatively minimal impact on the resulting ECM ultrastructure [140]. The hormonal regulation of tendon ECM also involves other pathways. Testosterone has been shown to promote tendon collagen content [141]. Higher collagen synthesis rates in male tendons correlate with circulating testosterone levels [131]. Furthermore, the Smad signaling pathway, which directly influences the expression of ECM-degrading enzymes (e.g., collagenases), is finely modulated by the endogenous sex hormone milieu. Estrogens generally exert a positive regulatory effect on the Smad pathway, often by upregulating its receptor components, whereas androgens tend to inhibit it through receptor downregulation [142,143].

### 5.3. Aging

Aging tendons are typically characterized by reduced collagen content, abnormal collagen composition, and increased GAG deposition. These alterations stem from cellular functional decline and ECM metabolic imbalance, ultimately leading to diminished biomechanical properties and impaired healing capacity [144]. Aging induces decreased collagen synthesis and increased degradation. On one hand, the number and function of collagen-producing TSPCs and tenocytes decline [145]. Concurrently, MMP-1, the rate-limiting enzyme that initiates the breakdown of native triple-helical type I collagen, is upregulated in dysregulated tendon ECM turnover [146]. This primary collagenolytic cleavage step is followed by increased activity of MMP-2 and MMP-9, which degrade denatured collagen fragments rather than intact fibrillar collagen, and thus participate in the downstream stages of ECM remodeling [147,148]. Alterations in collagen type ratios and structure also become abnormal, with aged tendons often exhibiting smaller, fragmented collagen fibers arranged in disordered patterns [124]. Some evidence suggests reduced collagen biosynthesis (type I and V collagen mRNA) alongside increased expression of type III collagen, associated with chronic degeneration [149]. Cellular senescence (producing reactive oxygen species, ROS) significantly accelerates AGE formation [150]. Due to the extremely long half-life of tendon collagen, AGEs accumulate persistently, severely disrupting ECM homeostasis. AGEs form via non-enzymatic reactions on lysine residues of collagen, increasing collagen stiffness. Furthermore, AGEs weaken collagen-proteoglycan interactions, impair cell adhesion and migration, and promote transglutaminase activity, further exacerbating cross-linking [151].

Studies indicate reduced protein levels of fibromodulin, mimecan, and asporin in aged tendons, alongside potential decreases in chondroitin sulfate, and dermatan sulfate content [152]. Reduced expression of glycoproteins such as lubricin, cartilage oligomeric matrix protein (COMP), and SPARC (a key matricellular protein involved in collagen fiber assembly) is observed, alongside decreased elastic modulus and failure stress [8,66]. In the tendons of aged rats, glycosaminoglycan content elevates, while the levels of elastin, tenomodulin, and certain matrix regulators, such as transforming growth factor-beta 3, are decreased [153].

### 5.4. Exercise

Exercise induces adaptive changes in tendons that counteract the negative effects of aging. These effects are primarily manifested in the promotion of tendon hypertrophy, alterations in ECM composition, such as increased proteoglycans and reduced AGEs and enhanced mechanical properties, including increased stiffness and elastic modulus [151]. In contrast, innate athletic ability exerts only a minimal influence on the tendon ECM [153]. Studies indicate that 12 weeks of daily 1-h incline treadmill running can successfully induce adaptive changes in tendons. At the mRNA level, expression of type III collagen is upregulated, while type I collagen expression remains unchanged. Insulin-like growth factor-I (IGF-IEa and IGF-IEb) expression is significantly increased, whereas expression of TGF-β1 and connective tissue growth factor (CTGF) decreases. Significant downregulation of mRNA for key non-collagenous components, fibromodulin and BGN, may reflect an optimization of matrix composition rather than pathological accumulation [154]. In patients with chronic Achilles tendinopathy, a single bout of acute exercise (a 1-h run) does not induce a significant inflammatory gene expression response in the affected tendon. Instead, it reveals an active ECM remodeling state characterized by upregulated collagen gene expression [155].

Tendon ECM undergoes complex remodeling in response to exercise. An optimal weekly running volume of 21–40 km is associated with the healthiest tendon ECM structure, characterized by favorable water content and collagen alignment. Both insufficient activity and excessive running volumes (>40 km/week) may increase the risk of developing a suboptimal ECM structure. Notably, even optimal running technique cannot fully mitigate the potential risks linked to excessive running volume [156]. Research indicates that running does not directly induce mechanical denaturation of collagen molecules in the Achilles tendon. Instead, collagen denaturation occurs during the post-exercise physiological remodeling phase and is mediated by MMP-driven degradation processes [21]. A single marathon or half-marathon is sufficient to trigger transient biochemical remodeling of the Achilles tendon ECM. These changes, detectable via ultrashort echo time magnetization transfer (UTE-MT) imaging, include disruption of collagen integrity, upregulation of macromolecular proteoglycans, and increased bound water content [157]. Completing a marathon also leads to an immediate, significant reduction in the anteroposterior diameter (thickness) of the Achilles tendon. This acute change likely reflects fluid efflux from the tendon ECM rather than a permanent alteration of the collagen architecture [158]. Long-term competitive distance runners exhibit significant structural adaptations in their Achilles tendon ECM, including reduced collagen organization, tendon thickening, and decreased echogenicity. Importantly, these changes are not associated with clinical symptoms, suggesting they may represent a protective remodeling response to repetitive high loads [159]. Similarly, long-term, high-intensity ballet training induces comparable structural alterations in the tendon ECM. While diffuse thickening likely represents a benign, adaptive response, the presence of focal moderate-to-severe hypoechoic regions may indicate a higher risk of progression to a pathological state [160].

Regarding experimental animal and methodological design, the running loads applied may be insufficient to disrupt tendon ECM homeostasis or trigger degenerative changes in certain animal models. Studies indicate that 16 weeks of high-speed treadmill running fails to induce pathological alterations in the ECM of rat Achilles tendons that are consistent with human Achilles tendinopathy. Therefore, high-speed treadmill running itself is not a reliable and reproducible animal model for inducing rat Achilles tendinopathy and its associated ECM pathology [161]. An 8-week period of moderate-speed (10 m/min, no incline) treadmill training also produces minimal effects on the rat Achilles tendon ECM [162]. Furthermore, studies indicate that uphill treadmill running cannot serve as a reliable animal model for simulating human Achilles tendinopathy pathology, including ECM degeneration [163]. From another perspective, these findings may suggest that regular, controlled running exercise could represent a physiological stimulus, as the applied models did not induce degenerative changes in the tendon matrix.

### 5.5. Genetics

Tendon ECM exhibits inherent variability in its intrinsic capacity to maintain homeostasis, resist injury, and undergo effective repair under mechanical loading [164]. Although present in low abundance within tendons, type V collagen plays a critical role in regulating the diameter of type I collagen fibrils and matrix assembly, thereby directly influencing the ultrastructure and mechanical properties of the ECM [165]. Among the related genes, Collagen type V alpha 1 chain (COL5A1) has garnered significant research attention regarding tendon ECM homeostasis and tendinopathies. COL5A1 encodes the α1 chain of type V collagen, which interacts with type I collagen to regulate collagen fibril diameter and matrix organization. Studies indicate a significant association between COL5A1 gene variants and tendon ECM health, as well as tendinopathy risk [166]. Specifically, the CC genotype at the COL5A1 rs12722 locus is associated with a higher risk of tendon rupture, whereas the TT genotype may confer a protective effect. In contrast, Collagen type XI alpha 1 chain (COL11A1, rs3753841and rs1676486) and COL11A2 (rs1799907) polymorphisms show no significant association with rotator cuff tendinopathy [167]. Furthermore, studies indicate that COL5A1 expression levels regulate the ultrastructure of collagen fibers in healing tendon ECM in a dose-dependent manner, differentially influencing the tissue’s final mechanical properties. Heterozygous knockout of this gene partially restored the stiffness of healing tendons, whereas homozygous knockout did not improve specific properties [47]. The impact of COL5A1 haploinsufficiency on tendon ECM involves a multi-layered, networked pathological process. It not only disrupts collagen fiber assembly directly but also triggers extensive ECM molecular remodeling and cellular responses by altering key matrix molecules and abnormally activating the TGF-β pathway [168]. Interestingly, the COL5A1 rs12722 CC genotype has also been associated with increased extensibility in knee extensor structures. These genetic variants are thought to alter the production of type V and type XI collagen, thereby jointly regulating collagen fibril organization within the ECM to influence tendon homeostasis [169]. This seemingly contradictory finding reflects the complex and context-dependent nature of soft tissue injury. COL27A1 encodes the α1 chain of type XXVII collagen. Its single nucleotide polymorphisms are associated with Achilles tendinopathy and may interact with COL5A1 variants [170,171]. Genes encoding major structural components such as type I collagen are likely so critical to function that harmful functional variants are rarely observed in populations, likely due to natural selection.

The promoter region polymorphism (5A/6A) of the MMP3 gene is associated with the risk of Achilles tendinopathy. This variation likely disrupts the balance between ECM synthesis and degradation by affecting enzyme expression levels, thereby contributing to degenerative changes in tendon ECM [166,172]. Regarding another MMP3 variant, the TT genotype and T allele of rs679620 were significantly more frequent in the uninjured control group than in the group with tendon pathology. This suggests that this genotype/allele may exert a protective effect by maintaining a more favorable balance in ECM synthesis and degradation, thereby reducing the risk of tendon degeneration. Interestingly, the relationship between this genotype and ligament injury follows an opposite pattern [166]. Furthermore, genes such as Actinin alpha 3 (ACTN3) may indirectly influence tendon health by altering the mechanical environment, whereas variants in COL1A1 and Growth differentiation factor 5 (GDF5) are more strongly associated with injuries in other connective tissues (Figure 3) [173,174,175].

## 6. Tendon ECM and Disease

### 6.1. Diabetes

Extensive preclinical and clinical evidence has established type 2 diabetes mellitus (T2DM) as a major contributor to tendinopathy [176]. Diabetes disrupts the hierarchical structure and histological morphology of collagen by inducing non-enzymatic cross-linking, thereby altering the molecular composition and renewal balance of the ECM, which impacts tendon homeostasis [177]. Diabetes disrupts the homeostasis of the connective tissue ECM through multiple intertwined pathways, including the formation of AGEs, oxidative stress, chronic inflammation, and impaired cellular function. This dysregulation leads to abnormal ECM composition, structural disorganization, diminished mechanical properties, and significantly impaired post-injury healing [178].

AGE-induced cross-linking alters collagen fiber ultrastructure, resulting in highly disorganized, twisted, and overlapping fibers while inhibiting their normal gliding [179]. By binding to the cell surface receptor for AGEs (RAGE), AGEs activate downstream signaling pathways that promote reactive oxygen species (ROS) production, primarily via NADPH oxidase (NOX) isoforms NOX1 and NOX4, which are key ROS-generating enzymes in tendons [180]. Furthermore, increased expression of MMP2, Tissue inhibitor of metalloproteinase 2 (TIMP2), the proinflammatory cytokine IL-6, and type III collagen contributes to dysregulated matrix remodeling [181]. AGEs deposited in the ECM also suppress mitochondrial function in tendon cells, impairing their proliferative capacity and thereby weakening the intrinsic repair of tissue and remodeling potential [182]. Through receptor-mediated signaling (primarily via RAGE), AGEs activate pathways such as Wnt/β-catenin, ROS, and p38/MAPK, which in turn inhibit the proliferation of TSPCs and promote their apoptosis and senescence [183,184]. Additionally, AGEs drive the aberrant differentiation of TSPCs toward osteogenic and chondrogenic lineages (abnormal ossification/chondrogenesis) rather than supporting their differentiation into healthy tenocytes [185]. In the context of pharmacotherapy targeting the ECM, prim-O-glucosylcimifugin represents a small-molecule drug candidate for repairing diabetic tendon injury. It acts by activating the AMPK signaling pathway, thereby rescuing TSPCs from senescence and functional decline under high-glucose conditions and restoring their capacity to synthesize a healthy ECM [186].

### 6.2. Hyperlipidemia

Hyperlipidemia, characterized by elevated low-density lipoprotein (LDL), is an independent risk factor for tendinopathy. It drives chronic inflammation, oxidative stress, and cellular dysfunction via low-density lipoprotein and its oxidized forms, leading to the disruption of tendon ECM homeostasis and structural damage, thereby increasing the risk of tendon injury and impairing its repair capacity [187]. Hyperlipidemia triggers chronic inflammation through multiple pathways. Oxidized LDL (ox-LDL) upregulates the expression of inflammatory cytokines, chemokines, and adhesion molecules. Itactivates the release of inflammatory mediators from immune cells and resident tendon cells [188]. As a ligand, ox-LDL binds to the receptor for advanced glycation end products, thereby activating the NF-κB pathway to induce innate immune responses and exacerbate inflammation [189]. Elevated cholesterol also impairs tendon-derived stem cells (TDSCs), inducing their apoptosis and autophagy and thereby compromising the cellular repair functions essential for ECM maintenance [190].

Of course, the impact of hyperlipidemia on tendons is a long-term dynamic process, which implies the existence of early compensatory molecular mechanisms. Studies have found that hyperlipidemia did not disrupt the ECM structure of the porcine rotator cuff tendon but rather triggered ECM remodeling. The core mechanism involves the upregulation of the transcription factor YY1, which promotes the gene transcription of type I collagen and inhibits MMP3, leading to the inactivation of downstream collagenases such as MMP1, MMP8, and MMP9, thereby protecting collagen from degradation. Simultaneously, under hyperlipidemic conditions, the transcription factor twist family bHLH transcription factor 1 (TWIST1) exists exclusively in the form of a TWIST1-E12 heterodimer, which drives collagen cross-linking and maturation [191].

### 6.3. Inflammation

Chronic tendinopathy is an active, inflammatory mediator-driven degradation process rather than a passive degenerative condition. Multiple inflammatory factors collectively drive tendon structural disruption, loss of mechanical properties, and pain by promoting ECM degradation, altering collagen synthesis, and facilitating abnormal vascular and neural ingrowth [192]. The process is initiated by the recruitment and phenotypic polarization of immune cells, coupled with the dysregulation of local cellular communication networks. The chemokine C-C motif chemokine ligand 4-like 2 (CCL4L2) acts as a critical molecular link between macrophage-mediated inflammation and failed tendon repair. It promotes macrophage polarization toward a pro-inflammatory M1 phenotype, increasing the proportion of M1 macrophages and transmitting inhibitory signals to TSPCs, ultimately impairing their differentiation capacity [24].

Activated immune cells release core effector cytokines, among which IL-1β, IL-17A, and IL-6 are particularly pivotal. As a key inflammatory amplifier, IL-1β dysregulates tendon ECM turnover: it shifts the balance between matrix metalloproteinase-mediated degradation and TIMP-mediated inhibition, increases the content of both type I and type III collagen with an elevated type III/type I ratio, and thereby drives ECM structural disorganization and fibrosis. The production of mature IL-1β is triggered when genomic DNA and cellular debris released from necrotic tendon cells activate the AIM2 inflammasome in macrophages, leading to caspase-1 activation and the cleavage of pro-IL-1β [193,194]. IL-17A is another core inflammatory mediator driving pathological ECM remodeling in early tendinopathy [195]. In an unloaded ex vivo rats tail tendon model, IL-17A treatment induces severe disintegration and disruption of the tendon ECM. It potently upregulates inflammatory mediators such as IL-6 and C-X-C motif chemokine ligand 1 (CXCL1), as well as the transcription factors NFKB inhibitor zeta (NFKBIZ) and zinc finger CCCH-type containing 12A (ZC3H12A)—responses not elicited by TNF-α [196]. Furthermore, IL-6 signals acts on reparative fibroblasts (CD90+) in the tendon epitenon/paratenon through classical or trans-signaling pathways. This activates the fibroblasts, promoting their migration and proliferation toward the injury site. At the site, these activated fibroblasts secrete excessive MMPs and alter the collagen synthesis profile, creating an imbalance between ECM degradation and synthesis, which ultimately leads to collagen disorganization and loss of tissue mechanical properties [197].

## 7. Therapeutic Strategies Influencing Tendon ECM

### 7.1. Biologic and Injectable Agents

In the field of biologics, ECM components have gained research interest as potential therapeutic strategies. A notable example is CTHRC1, a novel matrix-derived protein that represents a promising biologic candidate. It alleviates tendinopathy, promotes tendon healing, and restores mechanical properties in animal models by activating the EGFR/MAPK signaling pathway to enhance TSPC function. This provides direct evidence for developing protein-based therapeutics that modulate the ECM microenvironment to stimulate endogenous repair [121]. In addition to protein-based biologics, collagen-based medical devices represent another important category of ECM-targeted therapeutics for tendinopathy. MD-Tissue, an injectable medical compound mainly composed of swine-derived type I collagen, functions as a mechanical scaffold that triggers mechanotransduction responses in human tenocytes via the actin cytoskeleton. It promotes type I collagen synthesis and cross-linking maturation, enhances focal adhesion formation and cell migration, and thereby remodels ECM homeostasis to facilitate tendon tissue repair, offering a novel regenerative and rehabilitative approach for tendinopathy management [198]. Furthermore, in tendinopathy, ECM degradation generates bioactive fragments, including matrikines and matricryptins, which originate from proteins such as type IV collagen, periostin, SPARC, elastin, and hyaluronic acid (HA). Owing to their diverse signaling activities, these fragments hold diagnostic and therapeutic potential for tendinopathy [199]. For instance, studies demonstrate that injections of HA with varying molecular weights can improve the viscoelastic properties of tendon tissue and promote functional ECM deposition. Injectable collagen-based medical devices such as MD-Tissue also facilitate tendon repair and healing via mechanical stimulation of tenocytes [198,200,201].

### 7.2. Gene Editing

In the realm of gene editing therapy, research has shown that targeting the mRNA of SPP1 (osteopontin), a key extracellular matrix-associated protein secreted by macrophages, can effectively mitigate inflammatory adhesions following tendon injury. Under pathological conditions, the SPP1/CD44/AKT signaling axis drives fibroblast activation, leading to their differentiation into myofibroblasts and excessive deposition of type I and III collagen. Employing the CRISPR-associated protein 13 (CRISPR-Cas13) system to specifically recognize and cleave SPP1 mRNA has successfully modulated the local immune microenvironment and pathological ECM deposition in animal models. This presents a highly promising gene biotherapeutic strategy that targets ECM-related signaling to treat challenging conditions such as tendon adhesions [202].

### 7.3. Functionalized Composite Scaffolds

Beyond conventional biomimetic scaffolds, functionalized composite scaffolds have gained considerable attention in tendon tissue engineering and regenerative medicine, with ECM components playing a central role [203,204,205]. For example, a “book-like” bilayer scaffold designed for rotator cuff tendon-bone repair employs decellularized tendon ECM as its structural foundation. Its upper and lower surfaces are respectively functionalized with tannic acid-magnesium and tannic acid-zinc ion networks to enable spatially targeted therapy. In a rat rotator cuff tear model, this scaffold exerted synergistic anti-inflammatory and osteogenic effects, effectively promoting structural remodeling and mechanical recovery of the tendon-bone interface [206]. A similar decellularized tendon matrix graft further enhanced tendon healing in rats when combined with immobilized bone morphogenetic protein 12 (BMP-12) [207].

In another approach, type I collagen was coated onto aligned polylactic acid (PLA) electrospun fibers to improve biocompatibility and control drug release. This multifunctional composite scaffold concurrently loads with the anti-fibrotic agent rolipram, achieving sustained drug elution and supporting tendon cell adhesion, infiltration, and expression of functional ECM markers (type I collagen, tenomodulin) in vitro [208]. Furthermore, a decellularized hyaline cartilage graft (dLHCG) scaffold, rich in type II collagen, proteoglycans, and fibronectin, is developed to mimic the transitional ECM of the tendon-bone interface. It not only efficiently carries stem cells but also guides their differentiation toward tenogenic and osteogenic lineages, significantly enhancing functional healing in a rat rotator cuff model [209].

Studies have also identified periostin as a critical ECM protein for tendon development and stem cell maintenance. When combined with a scaffold that replicates the parallel alignment of native tendon ECM, periostin promotes TSPC proliferation, counters senescence, and enhances tenogenic differentiation, achieving structural and functional tendon regeneration in mice and offering a translatable strategy for repairing tendon defects [30]. An injectable, tendon-derived decellularized ECM hydrogel acts not only as a biomimetic carrier but also actively modulates the immune microenvironment by driving macrophages toward a pro-repair M2 phenotype. When used to deliver tendon stem cell-derived exosomes, the hydrogel synergistically enhances anti-inflammatory and regenerative outcomes in animal models, providing an integrated, microenvironment-targeted strategy for tendinopathy [31]. Similarly, an injectable small-intestinal submucosa (SIS)-ECM hydrogel loaded with the anti-inflammatory compound gastrodin effectively treated rat Achilles tendinopathy. Gastrodin suppressed local inflammation (reducing IL-6, elevating IL-10) via NF-κB inhibition, while the SIS hydrogel provided a biomimetic ECM microenvironment. Together they promoted orderly collagen remodeling and restored near-normal tendon biomechanics [210].

It is worth noting, however, that in three-dimensional tissue-engineered tendon models, excessive activation of endoplasmic reticulum-associated autophagy can alter collagen fiber diameter distribution and significantly impair key mechanical properties, such as ultimate stress and strain [211].

### 7.4. Bioactivity of ECM Healing

Recent studies have leveraged the bioactivity of tendon ECM to enhance healing at the tendon-bone interface, addressing the clinical challenge of poor repair outcomes. A novel enzymatic decellularization method applied to human Achilles and patellar tendons better preserves key native tendon ECM proteins, particularly members of the transforming growth factor-β family. The decellularized tendon was further digested with collagenase to produce a moldable, viscoelastic “DTM paste”, which can be intraoperatively shaped and applied to the injury site, showing promise for the treatment of refractory rotator cuff tears [212]. Tissue-engineered patches based on autologous tendon ECM have also emerged as a viable repair strategy. Such a patch is not a traditional biomimetic or decellularized scaffold, but a functional composite consisting of autologous tendon tissue particles embedded in a fibrin glue carrier. In a rabbit model of full-thickness rotator cuff defect, a patch fabricated from minced long head of biceps tendon-derived ECM components within a hydrogel carrier was implanted. After three months, the regenerated tissue exhibited significant improvement in both the structure and composition of the ECM [213]. It should be noted, however, that the specific source, processing, and properties of the patch material are critical. Some biomaterials designed to “augment” repair may interfere with or even disrupt the native tendon ECM during early healing in humans, and carry unpredictable risks of immune reaction [214].

Looking forward, tendon ECM can also be harnessed to actively guide a “bottom-up” self-assembly and regeneration process. By precisely mimicking the physical and biochemical microenvironment of the ECM, functional tendon organoids that recapitulate native tendon ECM characteristics, cellular composition, and strong regenerative potential can be scaled up in vitro. This approach provides a highly promising “off-the-shelf” graft strategy for the repair of clinically significant tendon defects (Table 1) [215].

## 8. Conclusions

The tendon ECM is a dynamic, information-rich, and highly regulatable biological system. It provides tendons with their exceptional mechanical properties through precise hierarchical assembly and a complex molecular composition, serving as a critical hub for converting mechanical signals into biological responses and cellular microenvironmental regulation. Its homeostasis is finely tuned by physiological loading, systemic cues, and individual factors, yet can be disrupted by the chronic inflammation and pathological remodeling driven by metabolic diseases, ultimately leading to tendinopathy.

Future research can delve deeper into the constituents of the tendon ECM, particularly specific types of collagens, non-collagenous proteins, and their associated molecular pathways. From a physiological perspective, further investigation is warranted into the spatiotemporal dynamics of endogenous tendon repair, including the sequential remodeling of ECM components across injury phases, the activation and recruitment kinetics of the TSPC niche, and the mechanobiological coupling between mechanical loading and repair programming. In particular, the multifaceted roles of small matrix regulatory proteins (such as the SLRP family, matricellular proteins, and glycoproteins) and GAGs during repair processes merit focused investigation. These molecules not only act as structural organizers that guide collagen fibril assembly, cross-linking maturation, and hierarchical architecture formation, but also function as bioactive mediators that sequester and present growth factors, modulate inflammatory macrophage polarization, and mediate stem cell–microenvironment crosstalk. Furthermore, the roles of the ECM and its encoding genes in adapting to mechanical forces and in various disease contexts warrant further exploration to identify optimal therapeutic and exercise protocols. Additionally, bioactive components derived from the ECM can be exploited to engineer advanced functional composite scaffolds or be applied directly to enhance tissue repair.

In summary, viewing the tendon ECM as a decodable, targetable, and mimickable integrated system is key to achieving functional regeneration. The path forward lies in interdisciplinary convergence, translating fundamental biological understanding into innovative therapeutic applications, offering promise not only for treating tendon disorders but also providing a paradigm for understanding the homeostasis and repair of other fibrous connective tissues.

## Figures and Tables

**Figure 1:**
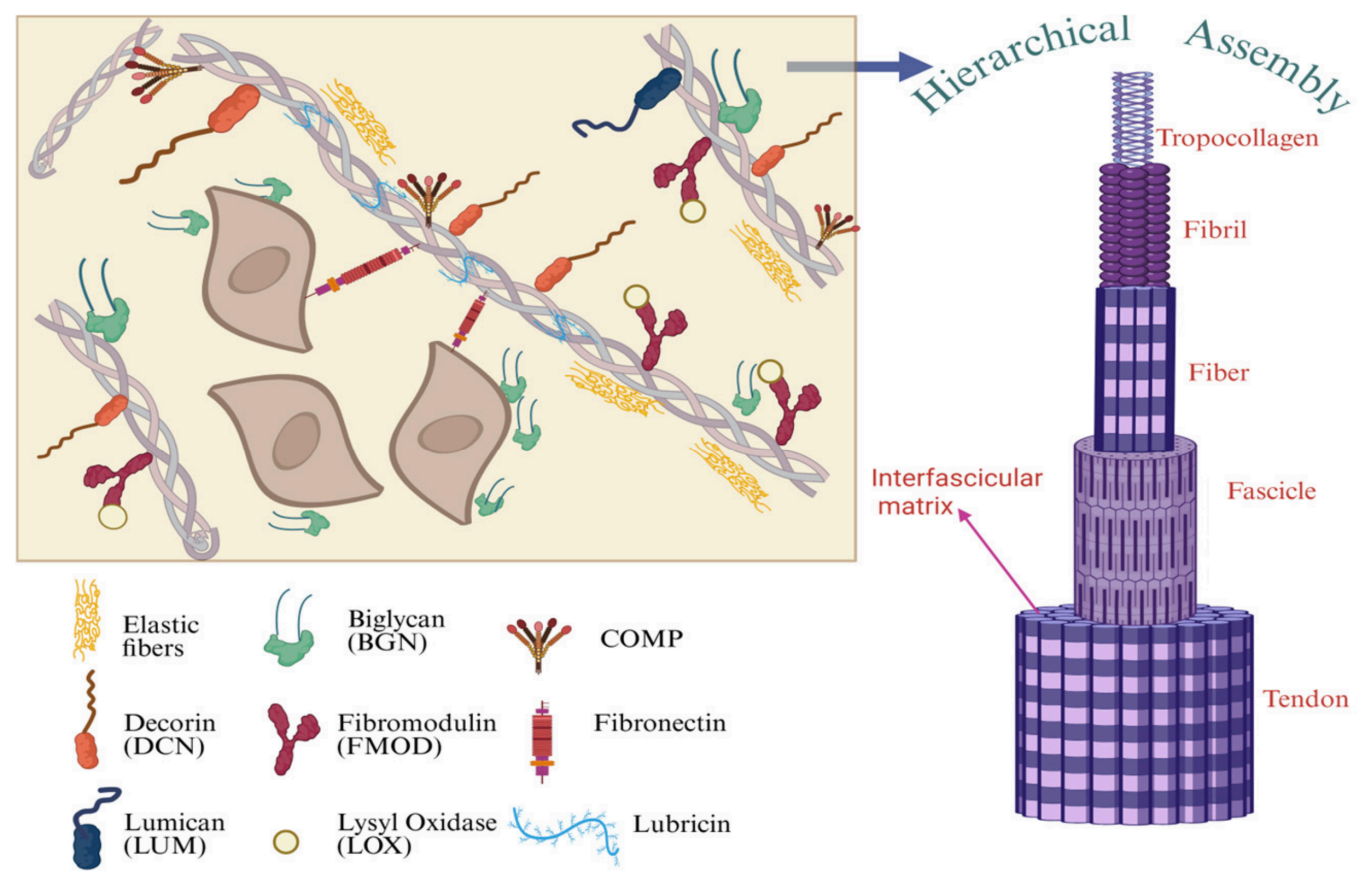
Molecular composition and hierarchical assembly of the tendon ECM. The tendon ECM is predominantly composed of type I collagen (~95% of total collagen), which assembles hierarchically from triple-helical tropocollagen molecules into fibrils, fibers, and fascicles that are in turn bundled to form the whole tendon. This assembly is stabilized by enzymatic cross-linking, primarily catalyzed by lysyl oxidase (LOX), which is guided by the small leucine-rich proteoglycan fibromodulin (FMOD). Other small leucine-rich proteoglycans, including decorin (DCN), biglycan (BGN), and lumican (LUM), regulate fibrillogenesis. DCN primarily regulates lateral fibril growth, BGN is more implicated in the early stages of fibril nucleation and organization, influencing fibril diameter and spacing, and LUM contributes to fibril assembly and spacing. In addition to collagens and proteoglycans, the tendon ECM contains elastic fibers, which provide tissue resilience, and a diverse set of glycoproteins (COMP, fibronectin, tenascin-C, and lubricin) that mediate matrix organization, cell–matrix adhesion, and interfascicular lubrication. Tenocytes are embedded within this matrix and extend cytoplasmic processes that contact the surrounding collagen fibers.

**Figure 2:**
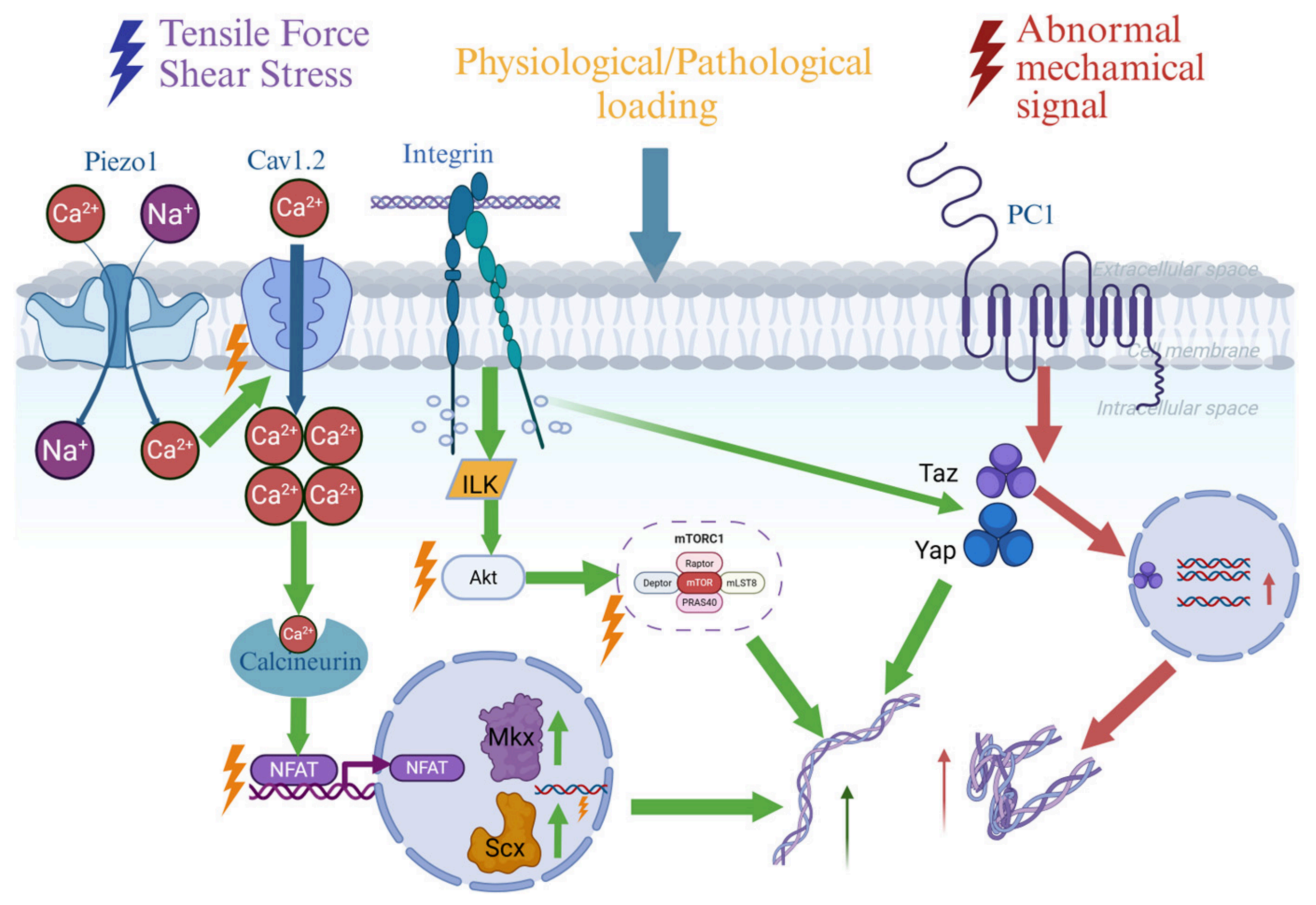
Mechanotransduction pathways regulating tendon eECM homeostasis and pathology. This schematic illustrates how tendon cells convert mechanical signals into biochemical responses that control collagen synthesis, assembly, and pathological remodeling. Under physiological loading, shear stress and tensile forces are sensed by mechanosensors such as Piezo1 and integrins. Piezo1 activation induces calcium influx, which activates the calcineurin/NFAT pathway to upregulate tendon-specific transcription factors (Mkx and Scx) and collagen synthesis. Concurrently, integrin engagement activates the ILK/Akt/mTOR signaling axis, promoting collagen fiber assembly and ECM deposition. Under abnormal mechanical signals, the mechanosensor Polycystin-1 (PC1) activates the Yap/Taz transcriptional co-activators, driving a pathological program that leads to ectopic ossification. Together, these pathways depict the critical “mechano-chemical-biological” cascade that maintains tendon ECM integrity under load or leads to dysfunction when dysregulated.

**Figure 3:**
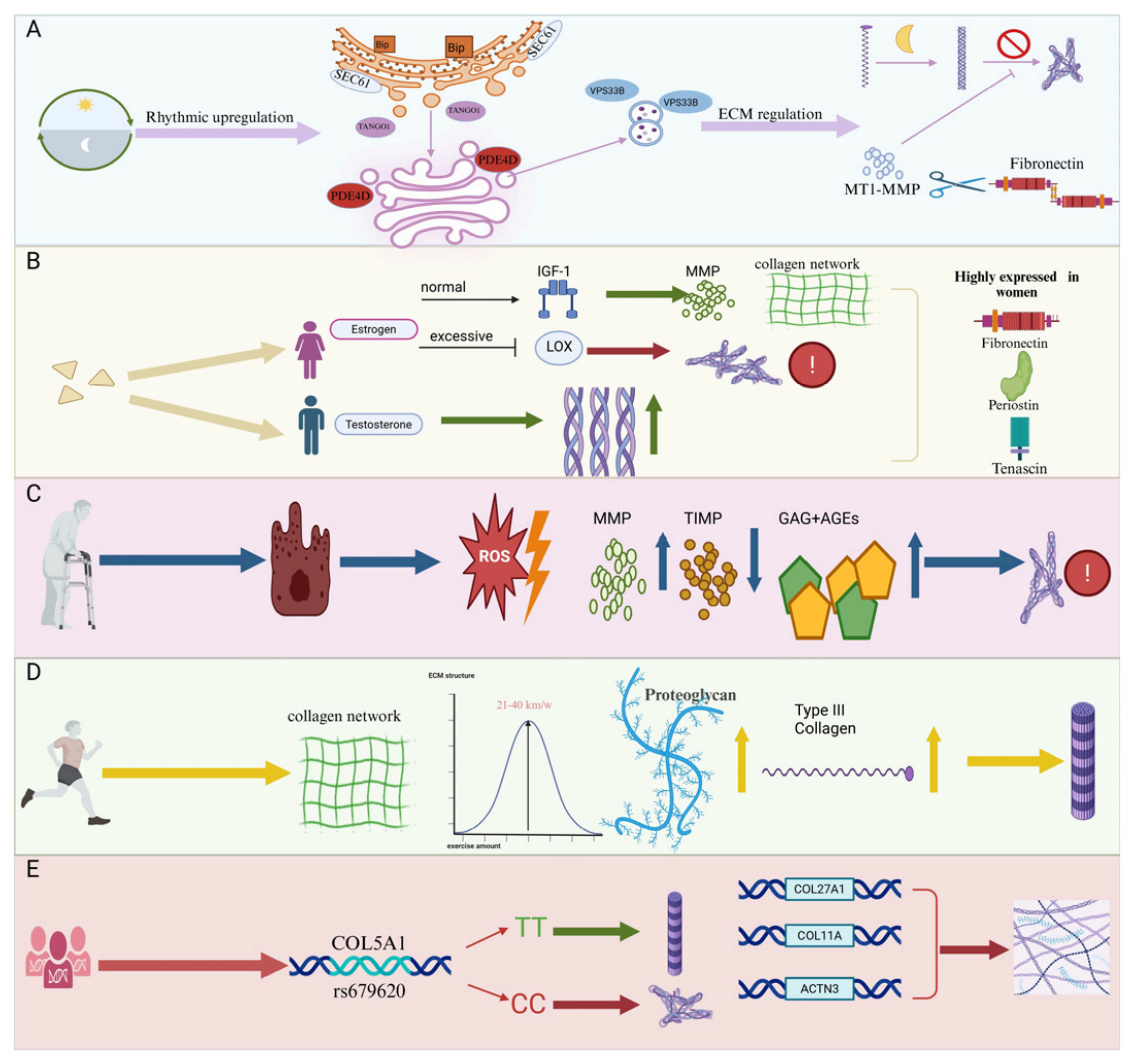
Factors Influencing Tendon ECM Homeostasis. (**A**) Circadian Rhythm: The molecular clock regulates nocturnal collagen I synthesis via rhythmic expression of secretory machinery (SEC61, TANGO1) and chaperones (BiP). Rhythmic Mmp14 prevents excessive collagen cross-linking, and clock-controlled Grem2 inhibits BMP signaling to protect against calcification. (**B**) Hormones: the effect of estrogen is concentration-dependent. It supports collagen synthesis, but at high levels inhibits LOX, reducing stiffness. Testosterone promotes collagen content. Female tendons have elevated repair proteins (Fibronectin, Periostin, Tenascin C). (**C**) Aging: Aging disrupts homeostasis via reduced collagen synthesis, increased MMP activity, and AGE accumulation, leading to disorganized fibrils and weakened mechanics. (**D**) Exercise: Optimal loading (e.g., 21–40 km/week running) promotes adaptive remodeling, upregulating collagen III and IGF-IE while downregulating TGF-β1 and certain proteoglycans. (**E**) Genetics: Variants in structural genes (COL5A1, COL27A1) affect fibrillogenesis, while regulatory variants (MMP3) alter ECM turnover. ACTN3 variants influence the mechanical environment.

**Table 1:** Tendon ECM: therapeutic applications in advanced medicine.

Application Field	Specific Technology/Product	Key ECM Component/Target	Mechanism of Action	Ref.
Biologics	CTHRC1 protein therapy	CTHRC1 (matrix-derived protein)	Activating the EGFR/MAPK pathway to enhance TSPC function, alleviating tendinopathy	[121]
Biologics	Matrix-derived fragment therapy	Matrikines/Matricryptins (from collagen IV, periostin, etc.)	possessing diverse signaling activities	[199]
Biologics	Hyaluronic acid injection	Hyaluronic acid of varying molecular weights	Improving tendon tissue viscoelasticity and promoting functional ECM deposition	[200,201]
Gene Editing	CRISPR-Cas13 targeted therapy	SPP1 (osteopontin) mRNA	Modulating the SPP1/CD44/AKT axis, reducing inflammatory adhesions and pathological ECM deposition	[202]
ECM-based Scaffolds	“Book-like” bilayer scaffold	Decellularized tendon ECM + Tannic acid-Mg/Zn networks	Enabling spatially targeted anti-inflammatory and osteogenic synergistic effects	[206]
ECM-based Scaffolds	Decellularized tendon matrix graft + BMP-12	Decellularized tendon matrix + BMP-12	Enhancing tendon healing	[207]
ECM-based Scaffolds	Collagen-coated aligned PLA fibers	Type I collagen + poly (lactic acid) fibers	Improving biocompatibility and controls drug release	[208]
ECM-based Scaffolds	Decellularized hyaline cartilage graft (dLHCG)	Type II collagen, proteoglycans, fibronectin	Mimicking transitional ECM and guiding stem cell tenogenic and osteogenic differentiation	[209]
ECM-based Scaffolds	Periostin-functionalized scaffold	Periostin + aligned biomimetic scaffold	Promoting TSPC proliferation, countering senescence, enhancing tenogenic differentiation	[30]
ECM-based Scaffolds	Injectable tendon-derived ECM hydrogel	Decellularized tendon ECM hydrogel	Driving macrophages to a pro-repair M2 phenotype, modulating the immune microenvironment	[31]
ECM-based Scaffolds	SIS-ECM hydrogel + Gastrodin	Small intestinal submucosa (SIS) ECM + Gastrodin	Suppressing inflammation via NF-κB inhibition, providing biomimetic ECM with hydrogel	[210]
ECM Repair Strategies	DTM paste (moldable)	Enzymatically decellularized tendon ECM (preserves TGF-β)	Forming an intraoperatively shapable paste and applying it to the injury site	[212]
ECM Repair Strategies	Autologous tendon ECM patch	Autologous tendon particles + fibrin carrier	Developing functional composites and improving ECM structure and composition	[213]
Frontier Directions	Tendon organoids	Biomimetic ECM physicochemical microenvironment	Recapitulating the features and regenerative potential of native tendon ECM in vitro	[215]

## Data Availability

No new data were created or analyzed in this study. Data sharing is not applicable to this article.
